# 

**Published:** 1888-06-23

**Authors:** 


### The Student's Column.

### ROYAL COLLEGE OF SURGEONS.

The following gentlemen, having passed the necessary examinations for the diploma of Fellow, were at a meeting of the council admitted Fellows of the college and received their diplomas, viz. :—

### New Fellows Admitted.

Messrs. John Adams, M.D., Aberdeen, Ashburton, Devon ; Andrew Stanford Morton, M.B., C.M., Edinburgh, 26, Weymouth Street, W. ; John Arthur Kempe, L.R.C.P., London, Royal Alexandra Hospital, Brighton ; John Mackern, M.D., Cantab , 30, Cambridge Street, Hyde Park ; Thomas Frederick Pearce, L.R.C.P., London, 10, Montague Street, Russell Square, W.C. ; Henry Work Dodd, L R.C.P., London, 47, Ken ington Park Gardens, W. ; Charles Arthur Morris, M.B., Cantab , 33, Myddleton Square, E.C. ; Wheelton Hind, M.D., London, 8, Woodhouse Terrace, Stoke-on-Trent ; Edgar W lliam Willett, M.B., Oxon, 60, Welbeck Street, Cavendish Square ; Ernest Charles Arnold L.R.C.P., London, Hurst, Fairfield Road, Croydon ; George Lee Wells, M.B., and B S., London, 16, Heathcote Street, W.C. ; James Harry Ernest Brock, M.B., London, 115, Adelaide Road, South Hampstead ; Arthur John Drew, L.R.C.P., London, 62, St. John Street, Oxford ; Thomas Pugh Beddoes, L.S.A., 89, Lambeth Palace Road ; Franke Chamberlain Hart-Smith, M.B., London, St. Peter's Rectory, Bedford ; Hugh Armstrong, L R C.P., London, Aylestone Hill, Hereford ; Reginald Robert Whishaw, L.R.C.P., London, 17, Mount-road, New Brighton, Cheshire ; Henry Secker Walker, L R C.R., London, The Elms, Wakefield ; Reginald Francis Jowers, L.R.C.P , London, 27, Old Steyne, Brighton ; Henry Tonks, L.R.C.P., London, Parkwood Grange, Knowle, Warwickshire ; Kankai Totsuka, L.R.C.P., London, 65, Lambeth Palace Road ; Ernest Solly, M.B., London, Congleton, Cheshire ; Charles James Shields, M.B., Melbourne, 289, City Road, E.C. ; Arthur Sneyd Taylor, M.B , Cantab, Richmond House, Elliot Park, Blackheath ; Frederick Howard Taylor, M.B., London, 8, Pyrland Road, N. ; Alfredo Antunes Kanthack, L.R.C.P., London, 1, Bentley Villa, Church Grove, Hampton Wick ; Patrick Whyte Rattray, M.B. and C.M., Aberdeen, 7, Approach Road, London, E. ; David Reid M'Kinnon, M.B. and C.M., Aberdeen, Bethnall House, Cambridge Road, E. ; John Wychenford Washbourn, M.B., London, The Friars, Gloucester ; Herbert Fox, L.R.C.P., London, 3, Brambletye, Park Hill, Croydon ; Percival Allan Lloyd, L.R.C.P., London, 101, St. Mark's Road, North Kensington. Twenty-one candidates were referred.

**Special Notice to Newsagents, Advertisers, and the Trade.**

**CHANGE OF OFFICE AND PUBLISHER.**

Notice is hereby given that the Office of The Hospital will henceforth be at 38, Old Jewry (Cheapside end), E.C., to which address all communications should be sent. Mr. A. Doig has been appointed Advertising Agent, and Mr. J. H. S. Hanning, Manager. All Cheques and Post Office Orders should be made payable to J. H. S. Hanning, 38, Old Jewry, E.C. crossed Lloyds, Barnetts, and Bosanquets Bank (Limited).

**To Secretaries and Matrons.**

The Editor will be glad to have a copy of the Regulations, and also of every Report issued from the Press of Asylums, Hospitals, Convalescent and Nursing Institutions, as well as those of other Charities, so soon as they are published, and an early copy in duplicate of all appeals for funds. Notices of Annual and other Meetings, Festival Dinners, etc., will be welcomed. All such communications should be addressed to *The Editor, The Lodge, Porchester Square, London, W.* Any superintendent, secretary, matron, or other chief official who will regularly send such documents and information will be registered to receive free of cost a cover for binding The Hospital in half-yearly volumes on sending name and address to the publisher.

### AMUSEMENTS WITH PROFIT FOR ALL AGES.

**Rules.**—All answers must be addressed to the PRIZE EDITOR, THE HOSPITAL, 38, Old Jewry, Cheapside, London, E.C., not later than the *first* post on Thursday next. The full name and address must in all cases be given, but a *nom de plume* may be added for publication. Only one side of the paper must be written on.

### FOR MEN AND WOMEN.

### First Quarterly Poetical Competition.

Commenced May 26th, 1888; ends August 25th, 1888.

This quarter a PRIZE of ONE GUINEA will be given to the competitor who gains the highest number of marks for original poems, on any subjects suitable for insertion in this paper. All contributions sent in will be credited according to merit eighteen marks being the highest score for any one contribution. This competition to alternate weekly with the set acrostic competition.

### Acrostic Competition.

This quarter the original acrostic competition will be discontinued, and A PRIZE of HALF-A-GUINEA will be given to the competitor who gains the highest score for solution of acrostics set. *One mark* only will be given to the competitors who solve all the lights of each acrostic.

Exception will be made in the case of *quotation acrostics*, when *two marks* will be given, *one* for the correct solution of all *lights*, and *one* mark for the *source* of the quotations.

This week credit will be given for solution of

### No. 2.—Double Acrostic.

Two traits of life are here combined,
The diaper of romance's page;
The smiling and the weeping sage,
Own'd the twin touches of mankind.

Sweet Gray, did Eton boys indeed
Delight, as in thy page we read,
"To chase the rolling circle's speed"?

Fiercely the monster leapt,
Then awe-struck crouch'd at length,
And kissed her feet and lickt her hands:
"Beauty can master strength."

Fell demagogue! whose fierce command
Made fat the headsman's knife,
'Twas pity nerved a woman's hand
To drink thy forfeit life!

A parting kiss she gave,
A parting tear she wept,
But while the other clave,
She turned, and homeward stept.

Poor trait'rous wretch! thy awful doom
Wins warmest pity's sigh.
To see fair sons around thee starved,
Then starved, thyself to die.

No happy valley can his wishes bound,
Whose fancy other fields beyond hath found;
In other fields, he tempts the moral strife,
To make, so he protests, his choice of life.

### Answers.

No. 1.—DOUBLE ACROSTIC.

| | | | |
|---|---|---|---|
| 1. | C | orf | U |
| 2. | A | lcoho | L |
| 3. | L | ibert | Y |
| 4. | Y | ell | S |
| 5. | P | lu | S |
| 6. | S | ens | E |
| 7. | O | lympu | S |

Correct answers have been received from Ellice, K. M., Sycamore, Alice, Dodo, Rowdy Corner, Condorcet, Ruffles, Hesperornis, Alicia, Tinie, Reldas.

### THE WORD COMPETITION.

Third Quarterly Competition commenced May 5th, 1888; ends August 4th, 1888.

Three prizes of one guinea, 15*s.* and 10*s.* 6*d.* will be given each quarter to the three competitors who obtain the highest number of marks by making the largest number of words derived from the word or phrase set for anatomical dissection; that for this, the *seventh* week of the quarter, being

"EMIGRATION."

OBSERVE.—Proper names, abbreviations, foreign words, words of less than four letters, and repetitions are barred; plurals, and past and present participles of verbs are allowed. Nuttall's *Standard* dictionary only to be used.

N.B.—All words sent in must be arranged *alphabetically*, with correct total affixed.

### Results of Fifth Week.

*Coercion.*—Tinie (28), Patience (28), Reldas (27), Sym (26), Dollie (25), Lightowlers (24), Lauriston (24), Dodo (22), Puff (22), Edge (20), Carmen (19).

### Notices to Correspondents.

*S. M. W.*—You should have been credited with 12 marks for No. 1 Original Poem last week.

### THE CHILDREN'S CORNER.

PRIZES.

FOR MOST CORRECT ANSWERS TO PUZZLES.

This Commenced October 1st, 1887.

One Pound a Month.

A PRIZE OF THREE GUINEAS

to the Competitor who shall have sent in the largest number of correct or best answers during the twelve months.

### Puzzles.—Third Week.

No. 11. LOGOGRIPH.—I am a word of eleven letters: my 9, 5, 11, is a number; my 7, 1, 4, 10, 6, to throw; my 9, 10, 2, 9, 5, a vegetable; my 1, 4, 3, 11, is what we now do; my 8, 9, 5, a weight, and my whole the name of a great traveller.

No. 12. HISTORICAL MENTAL PICTURE.—In a presence chamber a Queen is standing, surrounded by the chief officers of state. Two of those present—one a young nobleman of great bravery, generosity, and genius, and the other an experienced politician—are debating. In the heat of the argument, the young noble turns his back on the Queen, at which she is so incensed that she boxes his ears. Give the names of the Queen and the two courtiers.

No. 13.—CHARADE.

My *first* is swift and dangerous too,
My *next* in earth is hid from view,
My *whole* is often used to feed
The aged and the invalid.

No 14.—ENIMA.

Ere Adam was, my early days began,
I ape each creature and resemble man;
I gently walk o'er tops of tender grass,
Nor leave the least impression where I pass.
Touch me you may but I can ne'er be felt,
Nor ever yet was tasted, heard, or smelt.
Yet seen each day, if not, be sure at night,
You'll quickly find me out by candlelight.

### Results of First Week Puzzles.

No. 1. GEOGRAPHICAL CHARADE.—10 S E=Tennessee.

No. 2. TRANSPOSITION.—*Inch* transposed becomes *Chin*.

No. 3. DIAMOND PUZZLE.

```
      M
    T I N
  M I N E S
M I N E R V A
  H E R O N
    I V Y
      A
```

No 4. ENIGMA.—Lark-spur=Larkspur.

No. 5. CONUNDRUM.—When Algiers was ruled by Deys (days) and Malta by Knights (nights)

*All right.*—Hercules, Thanet, A. C. K. Scrooge, Happy, Eliza, The Young Canadian, Judy. *Four right.*—Alsie, Plummy Fluff, Mia Cara, R.H.

### Notices to Correspondents.

All competitors to the *Children's Corner* must send in their *age*, as well as *full name and address.*

### Conditions.

I. Every paper sent in must be clearly written, and one side only must be used. All competitors must mark at the head of their papers the number of their competition.

II. Each paper must be signed by the author with his or her real name and address. A *nom de plume* may be added, if the writer does not desire to be referred to by us by his real name. In the case of all prizewinners, however, the real name and address will be published.

III. All communications relating to prize competitions should be addressed to "The Prize Editor, THE HOSPITAL, 38, Old Jewry, London, E.C." Competitors are requested not to include in letters, etc., to the Prize Editor communications on matters of other business. All letters must be fully prepaid.

IV. The Prize Editor of THE HOSPITAL is the sole judge of the competitions, and his decision is in all cases final and without appeal.

V. The Prize Editor reserves the right to publish any paper sent in, whether it receives a prize or not. He does not hold himself responsible for any MSS. sent, nor can he undertake to return rejected competitions.

VI.—All children resident at school are requested to forward their *home* address in addition to the school one, when entering for the "Children's Corner" Competitions.

**Notice to all Correspondents.**—N.B.—All letters referring to this page, which do not arrive at 38, Old Jewry, Cheapside, London, E.C., by the *first post on Thursdays*, and are not addressed PRIZE EDITOR, will in future be disqualified and disregarded.

No one will be permitted to win the Monthly Prizes twice in any one year, the Annual Prize being offered especially to prevent this.

